# Theory of mind: mechanisms, methods, and new directions

**DOI:** 10.3389/fnhum.2013.00413

**Published:** 2013-08-08

**Authors:** Lindsey J. Byom, Bilge Mutlu

**Affiliations:** ^1^Department of Communication Sciences and Disorders, University of Wisconsin-MadisonMadison, WI, USA; ^2^Department of Computer Sciences, University of Wisconsin-MadisonMadison, WI, USA

**Keywords:** theory of mind (ToM), social perception, cognitive simulation, simulated social interaction, social cognition

## Abstract

Theory of Mind (ToM) has received significant research attention. Traditional ToM research has provided important understanding of how humans reason about mental states by utilizing shared world knowledge, social cues, and the interpretation of actions; however, many current behavioral paradigms are limited to static, “third-person” protocols. Emerging experimental approaches such as cognitive simulation and simulated social interaction offer opportunities to investigate ToM in interactive, “first-person” and “second-person” scenarios while affording greater experimental control. The advantages and limitations of traditional and emerging ToM methodologies are discussed with the intent of advancing the understanding of ToM in socially mediated situations.

## Introduction

Every day, humans engage in a wide variety of social interactions to achieve a diverse set of social goals that include acquiring information, influencing a partner's behavior through, and maintaining emotional intimacy through sharing thoughts and feelings. Integral to an individual's success in these social encounters is his or her ability to reason about the thoughts, beliefs, and feelings of others to predict behavioral responses. This ability has been termed *theory of mind* (ToM; Premack and Woodruff, 1978; Baron-Cohen et al., 1985).

The social importance of ToM can be demonstrated by imagining social interaction without it. To demonstrate the difficulty of explaining human behavior without ToM, Baron-Cohen (1997) used the task of explaining the behavior of a man who walks into a room, looks around, and then simply exits the room. Plausible mentalistic explanations can be easily generated for this scenario (e.g., the man forgot why he entered the room, the man was looking for something in the room and discovered it was not there); however, developing plausible non-mentalistic explanations of the man's behavior is more difficult (Baron-Cohen, 1997). The difficulties in generating concise and probable non-mentalistic explanations for this set of simple behaviors illustrate, on a small scale, the confusion that might result from complex behaviors like deception, persuasion, or flirting in the absence of ToM. Indeed, recognition of ToM's importance for social functioning has sparked extensive research in clinical populations for whom challenges in social interaction are common, including individuals with autism spectrum disorders (Perner et al., 1989; Happé, 1994; Baron-Cohen et al., 1995, 1999, 2001; Hill, 2004; Losh et al., 2012), schizophrenia (Corcoran et al., 1995; Brüne et al., 2007; Champagne-Lavau and Stip, 2010; Couture et al., 2011; Hooker et al., 2011), and traumatic brain injury (Bibby and McDonald, 2005; Havet-Thomassin et al., 2006; Henry et al., 2006; Milders, 2006; Muller et al., 2010; Turkstra et al., 2004; Turkstra, 2008).

Given the importance of ToM in daily interactions and the prevalence of ToM deficits in some clinical populations, it is important for researchers to critically consider both the concept of ToM as well as tasks used to investigate it. Accordingly, the goals of this article are to (1) present a framework of mechanisms that allow humans to infer and reason about mental states in social interaction, (2) review the benefits and limitations of current behavioral tasks designed to test each mechanism, and (3) discuss potential new directions for studying and understanding ToM, with consideration of both the advantages and the limitations that these approaches offer over more traditional techniques. It is our position that the knowledge to be gained from the incorporation of these new methodologies may advance not only the understanding of how humans reason about the mental states of others, but may also further sciences devoted to improving or compensating for ToM impairments and artificial intelligence research that is focused on developing artificial models of human-like social processes.

## Mechanisms of ToM

**Figure 1:**
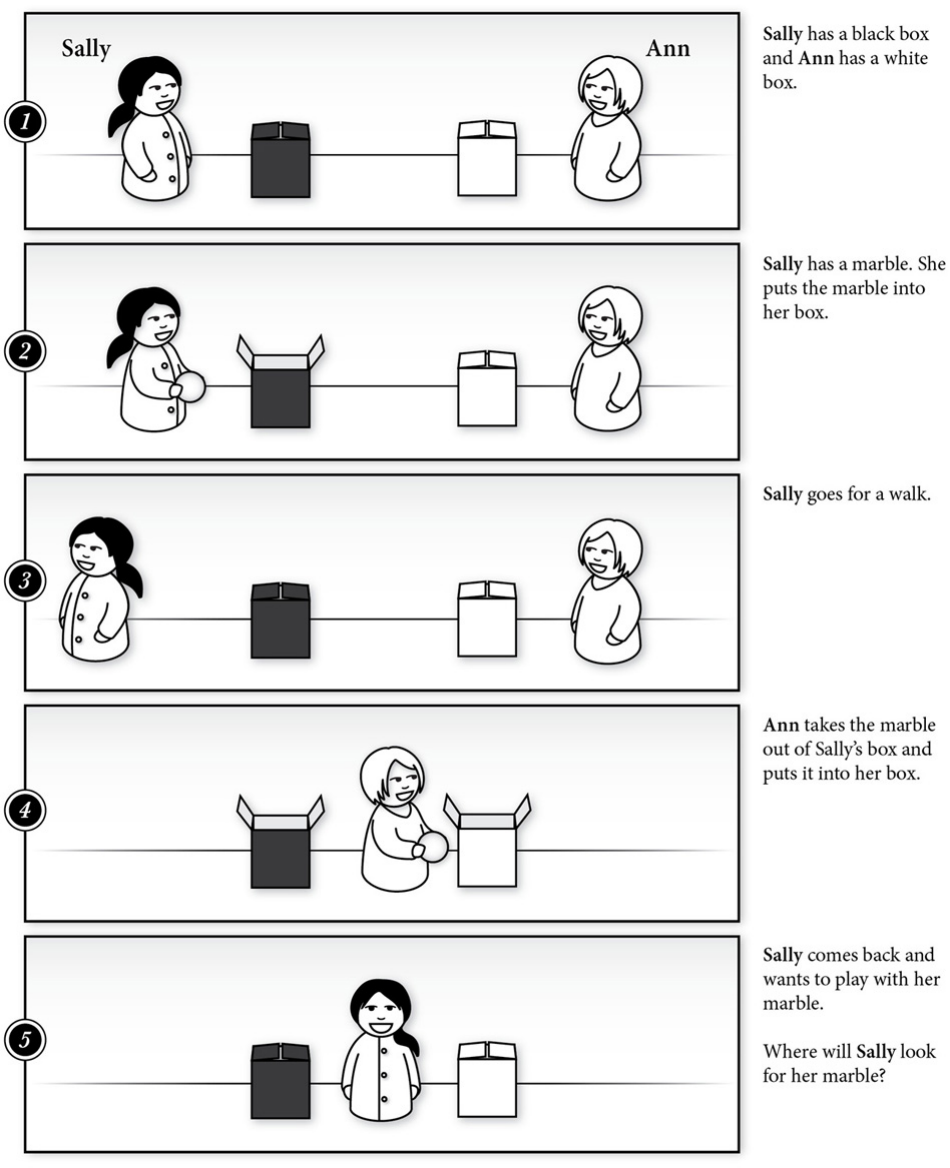
**A storyboard of the Sally-Ann or Location False Belief Task (Wimmer and Perner, 1983) used to test attribution of mental states to others in children**.

Since ToM research has been broad in terms of the interested disciplines, target populations and testing methodologies used, it is important to critically consider the concept of ToM, especially the ways in which our conceptualizations influence the course of ToM research. Developing a “working definition” of ToM will help guide research on not only the underlying network of skills that facilitate ToM, but may also provide insight into where breakdowns in ToM may occur. To begin forming such a definition, we posed the question, “How do humans accurately infer the mental states of others?” From this consideration, three components of interaction emerged as clues to ToM: (1) knowledge of the shared context, (2) perception of social cues, and (3) interpretations of actions, (See Table 1 and Figure 1). These components, and the experimental tasks developed to assess each, are reviewed below.

**Table 1:** **A summary of tasks used to test key mechanisms of Theory of Mind**.

**Mechanism**	**Type of task**	**Example task**	**Findings**
Shared world knowledge	Text-based tasks	Strange stories (Happé, 1994)	Individuals with autism have more trouble explaining the strange stories using mentalistic explanations than their peers without autism and those with mental disability (Happé, 1994; White et al., 2009).
	Non-verbal picture-based tasks	Character intention task (Sarfati et al., 1997)	Adults with TBI and schizophrenia are less accurate at choosing appropriate endings to comic strip stories where mental state attribution is needed (Sarfati et al., 1997; Havet-Thomassin et al., 2006)
Perceiving social cues	Facial emotion recognition	Reading the mind in the eyes task (Baron-Cohen et al., 2001)	Adults with TBI and autism have more trouble identifying mental states based on facial affect displays (Baron-Cohen et al., 2001; Havet-Thomassin et al., 2006; Turkstra, 2008).
	Facial/Vocal emotion recognition	The awareness of social inference test (TASIT; McDonald et al., 2006)	Adults with TBI and schizophrenia are less accurate at identifying facial emotions than healthy, uninjured peers (McDonald et al., 2006; Sparks et al., 2010).
Interpreting actions	False belief tasks	Reality unknown false belief (Wimmer and Perner, 1983)	Typically developing children begin to pass reality unknown false belief tasks around the age of 4 years (Wimmer and Perner, 1983); however, children with autism may fail to pass this task (Baron-Cohen et al., 1985).
	False belief tasks	Appearance reality false belief (Flavell et al., 1983)	Typically developing children begin to succeed on appearance reality tasks more frequently around the age of 4-years (Carlson et al., 2004).
	False belief tasks	Second-order false belief (Perner and Wimmer, 1985)	Typically developing children develop some competence in 2nd order false belief tasks between the ages of 6- and 7-years.

### Shared world knowledge

ToM, as one manifestation of cognition, is situated in the context of the surrounding world (Wilson, 2002). Consider, for example, the ToM demands implicit in a typical conversation. During a conversation, individuals must quickly infer their partners' thoughts, beliefs, emotions, and goals in order to formulate an appropriate response. As with other forms of joint action, making appropriate responses in conversation also requires the integration of cues from the conversational partner and the context, including prior world knowledge (e.g., amount of personal space with which a partner might be comfortable), knowledge about the relationship between individuals (e.g., how much disclosure is appropriate with a close friend vs. a co-worker), the goal of the interaction (e.g., what information is required to complete a joint task), and the conditions under which the conversation will occur (e.g., in a group setting) to make quick, on-line guesses about a partners' mental states (for review see Sebanz et al., 2006; Knoblich et al., 2011).

To investigate how shared world knowledge might facilitate ToM, researchers commonly present participants with short descriptions or picture sets of social scenarios and then ask participants to infer the mental states of characters or to predict characters' behaviors based on these inferred mental states (Happé, 1994; Havet-Thomassin et al., 2006). Happé's Strange Stories Task (1994) is one example of this type of task. The Strange Stories Task tests the ability to use prior world knowledge in order to understand several communication acts embedded in story situations, including faux pas, persuasion, pretending, and deception, and to accurately select the intended story interpretation.

Tasks like the Strange Stories Task, designed to assess an ability to reason about mental states through integration of shared world knowledge, are valuable in that they have been found to detect ToM deficits in both individuals with autism spectrum disorders and traumatic brain injury (Happé, 1994; Havet-Thomassin et al., 2006). However, it is important to consider that these tasks impose considerable cognitive demands while testing ToM, especially demands on working memory and, in the case of verbal or text-based tasks, linguistic processing. For example, in interpreting a story describing a scenario in which a character tells a white lie, participants must process the language of the story and hold the relevant information in working memory while that information is integrated with prior knowledge and an interpretation is selected. These demands become especially critical when investigating ToM of individuals who may have language disorders or deficits in working memory. Further, many of these tasks are passive and reflective in nature (e.g., Happé, 1994; Brüne et al., 2007), which may overestimate ToM ability in daily life. For example, tasks requiring individuals to make mental state inferences from described scenarios generally allow ample response time and do not typically require individuals to formulate appropriate responses as if they themselves were in the situation.

### Perceiving social cues

Another way in which humans infer the mental states of others is through the perception of various social cues. Though mental states are inherently cognitive phenomena, humans have a sophisticated repertoire of behaviors, including gaze cues, facial expressions, and vocal cues, through which they express their mental states.

The perception of gaze cues as a method in which humans infer mental states is one of the most studied mechanisms of ToM. Gaze cues signal the basic direction or object of one's attention (Bayliss et al., 2007; Frischen et al., 2007), and by following a partner's gaze, an individual is able to infer his or her partner's intentions (Baron-Cohen et al., 1995). An individual's ability to use gaze-based cues to infer the knowledge of others develops in childhood, and these cues have been found to outweigh deceptive verbal cues in experimental paradigms (Freire et al., 2004). Additionally, gaze cues in conversation allow an individual to monitor understanding of his or her message (Clark and Krych, 2004) and also to signal a desire for partner feedback or to surrender the speaking floor, (Kendon, 1967; Duncan, 1972; Bavelas et al., 2002). Further, speaker gaze cues may work to resolve linguistic ambiguities in non-literal language like sarcasm, as Williams et al. (2009) reported. For example, in Western cultures speakers tend to look away from their partners while making sarcastic comments, signaling that the speaker does not actually believe what he or she is saying (Williams et al., 2009).

Emotion recognition likewise has received much research attention as, like gaze cues, facial, and vocal emotional cues are valuable in the inference of mental states (De Sonneville et al., 2002). Typically, humans develop the ability to discriminate and perceive changes in facial expressions very early in life; however, the speed and accuracy with which children identify and match facial emotions continues to develop into adulthood (Barrera and Maurer, 1981; McClure, 2000). Both children and adults are generally quicker and more accurate in their identification of positive emotions like “happy” as compared to negative emotions like “sad” (De Sonneville et al., 2002). Humans also convey emotional content in aspects of vocal production including vocal intensity, prosody, quality, and speech rate.

Several tasks exist to study both gaze behavior and emotion recognition. Much has been learned about gaze behavior through the observation of dyadic interactions (e.g., Duncan, 1972; Clark and Krych, 2004) as well as through experimental manipulation of gaze cues (Baron-Cohen et al., 1995; Bayliss and Tipper, 2006). In a standard gaze perception task, individuals are shown a face with the eyes either oriented straight ahead or shifted in one direction. From these images, participants are asked to make inferences about the characters' intentions or mental states (Frischen et al., 2007). Similar tasks have been developed to assess comprehension of facial emotion (Ekman and Friesen, 1976). In one example, De Sonneville et al. (2002) presented participants with four faces, each of which portrayed a different emotion, and asked participants to determine whether or not a target emotion was demonstrated in one of the four foils. These authors also used a matching emotion recognition task, in which participants decided if two faces showed the same or different emotions (De Sonneville et al., 2002). Another method used to evaluate emotion recognition is to determine how accurately participants identify emotions from facial expressions with varying levels of subtlety. Thomas et al. (2007) employed a task in which participants viewed photographs of people portraying different degrees of various emotions, from very subtle anger to very obvious happiness, to detect the accuracy of participant emotion recognition. Vocal emotion recognition can also be evaluated with tasks similar to those designed to measure facial emotion. In these tasks, participants generally hear semantically neutral sentences with different forms of emotional prosody and are asked to identify the emotion of the speaker (Nowicki and Carton, 1993; Scherer and Scherer, 2011).

Like tasks requiring the use of shared world knowledge for ToM reasoning, social cue perception tasks have greatly contributed to what is understood about mental state reasoning. These tasks too, however, share limitations in their reflective, offline design, and limited ecological validity. Considering emotion recognition tasks, for example, participants are often presented with decontextualized images of faces (e.g., Bowers et al., 1999; Baron-Cohen et al., 2001) and are asked to either identify the emotion or to match it to a target (De Sonneville et al., 2002; Thomas et al., 2007). Additionally, even when stimuli are dynamic in the form of video clips (McDonald et al., 2006), participants are still given time to observe the stimulus, consider its properties, and make a judgment. In daily life, emotional displays, are fleeting but are rarely presented in isolation—redundant clues to mental states are presented in partners' words, faces, voices, and actions. This combination of presenting isolated social cues, which may underestimate actual abilities, and prolonged observation and thinking time, which may overestimate abilities, make it difficult to establish an accurate picture of the perception of social cues in everyday interaction. These limitations are of clinical importance because the ability to infer mental states from social cues has been commonly studied as a means to better understand the impact of social deficits on functioning in everyday life (Spell and Frank, 2000; Baron-Cohen et al., 2001; Croker and McDonald, 2005; Tonks et al., 2007; Turkstra, 2008; Zupan et al., 2009).

### Interpretation of actions

Research on the development of ToM has provided evidence that children as young as 6 months of age form expectations regarding how humans interact with other humans and inanimate objects (Legerstee et al., 2000). As humans, we generally believe that others act in ways that are consistent with their beliefs and goals (Heider and Simmel, 1944; Ajzen, 1991). Given this assumption, passively observing behavior can offer important clues regarding the intentions or beliefs of others. Several tasks have been developed to evaluate participants' abilities to infer mental states from behavior (Wimmer and Perner, 1983). For example, in a standard false-belief task (e.g., Wimmer and Perner, 1983), participants infer a character's belief based on the observations of her actions.

Tasks requiring the interpretation of actions are frequently used in developmental literature (Baron-Cohen et al., 1985; Luo, 2011; Scott et al., 2012), but studies of joint action have also provided insight into how humans interpret actions to infer the mental states of others (for review see Sebanz et al., 2006; Knoblich et al., 2011). For example, Sebanz et al. (2006) suggest that using gaze cues to infer what someone is attending to, as well as knowing what task the person is engaged in, helps observers to predict others' action goals. Supporting the relationship between observing actions and inferring mental states, Ramnani and Miall (2004) trained participants to perform a button-press task in response to visual symbols, with the symbol color indicating whether the participant, a partner, or the computer should respond. Neuroimaging data from this experiment suggested that predicting another's actions (i.e., predicting when a partner should act) activated neural regions important for ToM (Ramnani and Miall, 2004).

While tasks designed to test ToM through the observation of actions are inherently passive in nature, joint action tasks, like that used by Ramnani and Miall, have allowed for the study of ToM abilities in simulated interactions as opposed to simply reasoning about social scenarios as third-party observers.

## Emerging tools and methods to study ToM

The mechanisms discussed above allow individuals to draw on information about the actions, behaviors, and knowledge of others to make inferences about their thoughts, beliefs, feelings, and intentions. Individuals gather this information through reciprocal interactions and process it on-line to make ToM inferences and determine subsequent behavior. To study this interactive, on-line social-cognitive process, research on ToM has primarily used experimental paradigms that involve participants making ToM inferences from stimuli presented as static images (Baron-Cohen et al., 2001; De Sonneville et al., 2002; Mutlu et al., 2009), textual stories (Happé et al., 1998), or video vignettes (Turkstra, 2008) that provide an observation-based, reflective “third-person” understanding of ToM (Frith and Frith, 2006; Schilbach et al., 2012). Schilbach et al. (2012) argue that ToM—and social cognition in general—has fundamentally different motivational consequences and underlying neural processes when individuals are socially and emotionally engaged with others than when they are third-person observers. Interacting with others provides individuals with the ability to perform active conversational roles, which might include initiating or responding to episodes of interaction, rather than observing the interaction as a bystander. This active involvement also facilitates shared goals, intentions, and actions among the participants of the interaction, providing individuals with the ability to draw on firsthand experience in making ToM inferences.

Recent research has highlighted the limitations of experimental methods that provide “third-person” evaluations of social phenomena and has proposed a “second-person” approach to studying social cognition (Wilms et al., 2010; Duff et al., 2012; Risko et al., 2012; Schilbach et al., 2012). Risko et al. (2012) suggested that experimental paradigms designed to study social cognition form a continuum between simple, static representations of socially relevant stimuli and actual live social interaction or between “reel” and “real” instances of interaction. This continuum includes static schematic faces, dynamic schematic faces, static photographs of faces, static photographs of people in complex social scenes, dynamic images of people in complex social scenes, situations with the potential for real social interaction, and real social interactions. Studies that compare responses to stimuli from different sections of this continuum show significant differences. For instance, imaging studies show that direct gaze elicits significantly greater brain response than either gaze aversion or no gaze, but only when participants observe live stimuli and not when they observe static images (Ponkanen et al., 2010). Similarly, while the propensity to look toward another individual's eyes exists across the spectrum from schematic faces to dynamic social scenes, potential for actual social interaction significantly affects this propensity (Risko et al., 2012). These results highlight key limitations in the use of existing “third-person” paradigms in the study of ToM mechanisms and motivate the use of alternative approaches that afford studying “first-person” or “second-person” social cognition in “real” experimental paradigms.

### First- and second-person approaches to studying ToM

An emerging approach that seeks to build a first- or second-person understanding of ToM mechanisms and processes is the use of simulation-based computational methods such simulated social interaction (Blascovich et al., 2002) and cognitive simulation (Scassellati, 2002). These methods draw on advancements in computer sciences to employ complex computational systems that enable the simulation of embodied, situated interactions and thus the development of protocols with great experimental control and ecological validity.

#### Simulated social interaction

**Figure 2:**
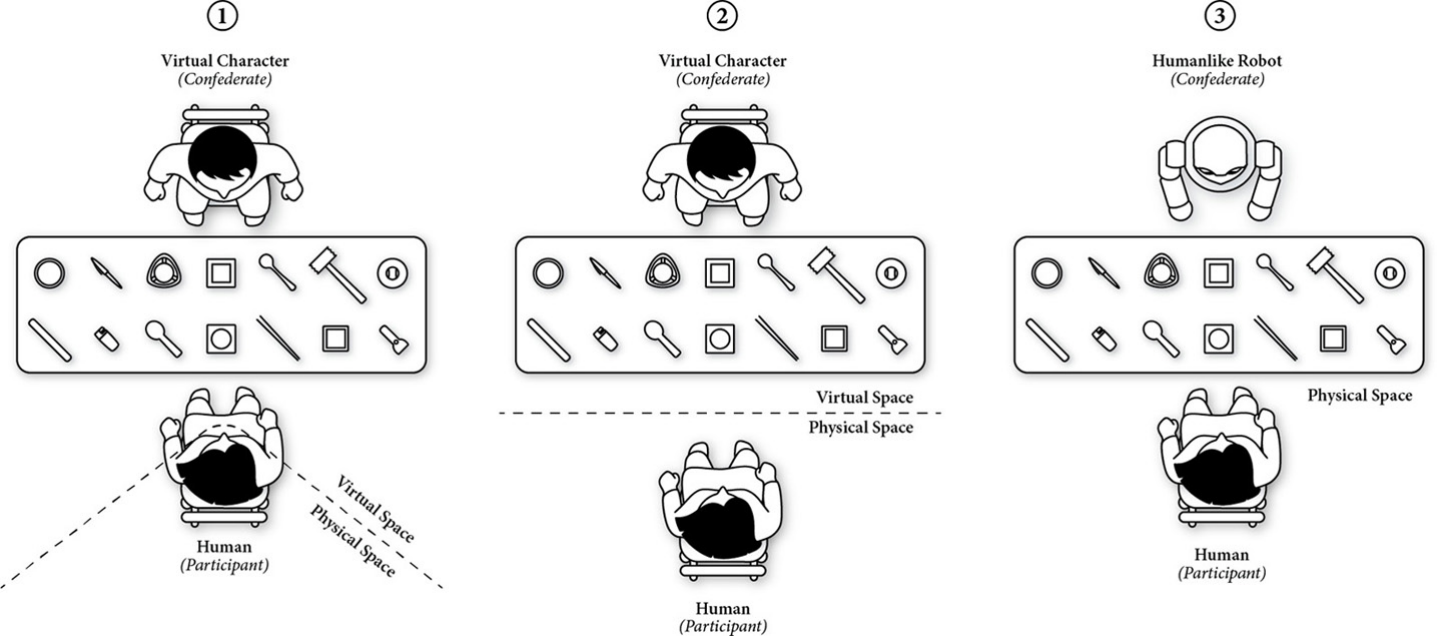
**Illustrations of three simulated experimental paradigms: (1) immersive virtual environments, (2) physical environment with a virtually simulated character, and (3) physical environment with a humanlike robot**.

Simulated social interaction involves generating social behavior in artificial agents such as virtual characters, which are often embedded in immersive virtual environments or as humanlike robots. Simulated social interaction offers greater experimental control and ecological validity than do traditional “reel” experimental paradigms (Loomis et al., 1999; Blascovich et al., 2002; MacDorman and Ishiguro, 2006; Mutlu et al., 2009; Wilms et al., 2010). In these experimental paradigms, participants interact with simulated others whose behaviors are precisely controlled to reflect experimental manipulations and who respond to changes in the participants' behaviors affording interactions that more closely resemble real-world interactions than static simuli do. These interactions might take place in immersive virtual environments, in the physical environment with a virtually simulated character (Pelphrey and Carter, 2008; Wilms et al., 2010), or in the physical environment with a humanlike robot (MacDorman and Ishiguro, 2006; Mutlu et al., 2009) (Figure 2).

**Figure 3:**
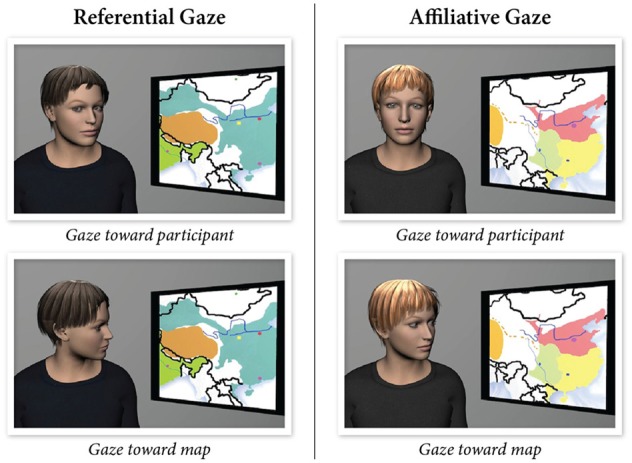
**The two types of gaze behaviors designed as experimental stimuli: referential (left) and affiliative (right) (Andrist et al., 2012)**.

Simulated social interaction offers a number of advantages over traditional experimental paradigms used for the study of ToM including precise control of experimental stimuli, interactive, dynamic social interaction, on-line processing and measurement. Thus, these methods more closely approximate the ToM demands of everyday interactions. Simulations of social stimuli follow computational representations of human behavior, which provide the experimenter with control parameters for the behavior or mechanism under study and the ability to create experimental manipulations that are impossible or infeasible for human confederates to perform. In a recent study, Andrist et al. (2012) developed a parametric model of gaze shifts and precisely manipulated how much the head of a virtual character aligned with its gaze target, creating two kinds of gaze shifts: affiliative and referential. Affiliative gaze shifts involved the virtual character aligning the head more toward the participant when it shifted its gaze in the environment, while referential gaze shifts involved the character aligning its gaze more with its gaze target (Figure 3). The results showed that affiliative gaze increased subjective evaluations of the character and the interaction, while referential gaze increased recall of information from the environment (Andrist et al., 2012).

The experimental paradigms involved in simulated social situations go beyond “presenting social stimuli,” but also offer reciprocal social interaction. For instance, Wilms et al. (2010) developed an experimental paradigm in which a virtual character shifted its gaze jointly with the participant using real-time eye-tracking input. Their results showed that establishing joint attention to an object of interest elicited greater activity in the brain (medial prefrontal cortex and posterior cingulate cortex) than did attending to the object non-jointly. The simulation approach also affords on-line processing and measurement of social interaction. For instance, another imaging study showed that a virtual character elicited greater brain activation in the superior temporal sulcus when it established mutual gaze with participants than it did when it averted its gaze as it passed by participants in the virtual world (Pelphrey et al., 2004), allowing researchers to measure an on-line neural response that could be captured by reflective approaches.

**Figure 4:**
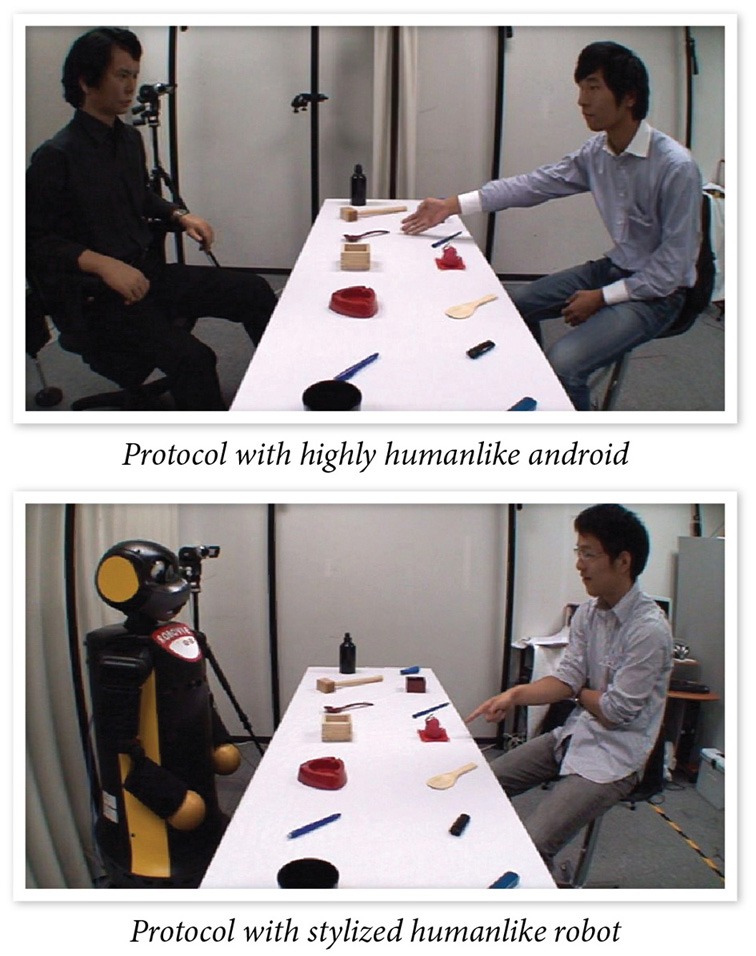
**Participants interacting with two humanlike robots in an interactive protocol designed to study how individuals might use nonverbal leakage to make ToM inferences (Mutlu et al., 2009)**.

The precise control, interactivity, and on-line processing afforded by this experimental approach offer greater ecological validity for the study of ToM and social cognition. In a study that embodies these characteristics, Mutlu et al. (2009) explored how leakage cues—non-verbal cues that individuals give off on their thoughts, beliefs, and intentions—presented by a robot might elicit ToM inferences in participants. Participants played a version of the 20-Questions guessing game with a humanlike robot in which the robot covertly picked one of the items that were laid on a table and the participants tried to guess which item the robot picked by asking questions that the robot could only answer with “Yes” and “No” (Figure 4). In half of the trials, the robot produced a brief gaze shift toward the item before answering the questions, producing a leakage cue, while it did not shift its gaze in the other half. Participants were able to identify the robot's pick with fewer questions and in shorter time when the robot produced leakage cues than they did when the robot did not leak information, suggesting that the participants used the socially relevant information to make ToM inferences and to more effectively narrow down the response options. Furthermore, the majority of participants did not report noticing leakage cues or using this information in the task, suggesting an implicit processing of such cues. This experimental paradigm offers the ability to precisely control the gaze cues presented by the robot, present these stimuli in an interactive, dynamic protocol, and support on-line processing toward shaping subsequent behavior in the interaction. The experiment also contextualized ToM processes in a simulated interaction that closely resembled face-to-face interaction and captured effects of ToM processes on objective measures of task performance.

#### Cognitive simulation

While simulated social interaction offers the ability to generate precisely controlled social behaviors in artificial agents and to create dynamic, interactive experimental scenarios, this approach relies primarily on pre-scripted and experimenter-controlled protocols—often referred to as Wizard-of-Oz studies (Dahlbäck et al., 1993). This approach does not afford the study of ToM processes in complex interactions such as joint action scenarios (Sebanz et al., 2006) that require all parties to engage ToM mechanisms in coordinating their actions toward a common goal. A complementary approach to simulated social interaction is cognitive simulation, which seeks to develop artificial representations of neurocognitive mechanisms such as imitation and perception of self, simulate them in artificial agents such as humanlike robots, and assess their functioning in enabling ToM inferences in human-agent interactions (Breazeal and Scassellati, 2002; Scassellati, 2002; Michel et al., 2004).

Building on simulation theory (Gallese and Goldman, 1998), cognitive simulation involves the robot establishing and maintaining representations of the mental states of its human counterparts by tracking and matching their states with resonant states of its own. These representations enable the robot to take the perspective of its human counterparts, make inferences about the human's goals, and learn from their actions. For instance, Trafton et al. (2005) developed a cognitive simulation that enabled the robot to simulate “alternative worlds” and assess propositions about these worlds in order to make inferences about the perspective of its human counterparts. A similar approach by Breazeal et al. (2006) involved the robot maintaining separate sets of “beliefs” in its belief system for itself and for its human counterparts. The separate sets of beliefs enabled the robot to identify differences in its beliefs from those of its human counterparts in order to plan actions that it might take or skills it might learn in order to establish a shared set of beliefs.

Examples of the cognitive simulation approach also include simulations of the motor-resonance mechanism (Blakemore and Decety, 2001) for enabling ToM inferences in artificial agents. These examples build on the finding that observing the actions of others elicits automatic activation of motor representations associated with these actions and enables predictions about action consequences. For instance, Gray et al. (2005) developed a system in which the robot parsed the actions of its human counterparts and matched them to actions in its own repertoire in order to make inferences about the goals of the participant. These inferences enabled the robot to perform task-level simulations and track the participant's progress in the task in order to anticipate the needs of its partner and offer help. A model developed by Bicho et al. (2011) extended this paradigm by including a mapping between observed actions and complementary actions required to successfully complete a task and enabled the robot to more effectively coordinate its actions with those of its human counterparts in the task.

The preceeding examples illustrate how cognitive simulation might complement the simulated interaction approach for studying ToM mechanisms by simulating ToM processes in artificial agents. When coupled, the two approaches promise two key methodological advances in the study of ToM. First, they help in assessing existing neurocognitive models of ToM mechanisms by computationally simulating them and observing system behavior in interactive situations. Second, they enable empirical studies to build new understanding of ToM processes in truly interactive protocols in which all agents—human or artificial—involved in the interaction employ ToM mechanisms. The coupling of the two approaches extends the methodological advantages of simulated social interaction by enabling not only on-line measurement of responses to social stimuli but also on-the-fly precise control over simulated cognitive mechanisms and social behaviors, thus affording even greater experimental control. The truly interactive setting of the protocols enabled by the coupling of these two approaches also improves their ecological validity.

### Example paradigms for studying ToM mechanisms

Recent research across many fields of social, cognitive, and computational sciences has developed first- and second-person experimental methods to study ToM mechanisms with the goal of gaining a better understanding of these mechanisms and designing artificial agents that effectively interact with people. The paragraphs below illustrate paradigms that study the three key ToM mechanisms described earlier.

#### Shared world knowledge

**Figure 5:**
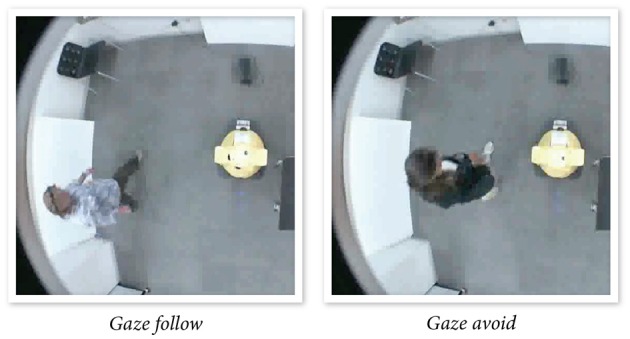
**Participants maintaining different amounts of distance between themselves and an “unlikable” robot in response to the robot's attempts to increase intimacy by following or avoiding the participants with its gaze (Mumm and Mutlu, 2011)**.

In making ToM inferences, individuals draw on a shared world knowledge to integrate information from various sources including resources in the environment, knowledge about social norms, the goals of the interaction, the relationship among interaction partners, and the participation structure of the interaction. Mumm and Mutlu (2011) investigated how the relationship between a humanlike robot and participants affected the participants' preferences for interpersonal distance, creating a “likable” or “unlikable” humanlike robot using verbal framing. Participants responded to the unlikable robot's attempts at increasing intimacy using mutual gaze by physically distancing themselves from the robot (Figure 5), while they did not change their proxemic behavior with a likable robot. The results suggest that the experimental protocol successfully established different relationships between the robot and the participant across the two conditions, which in turn shaped their preferences for interpersonal distance, enabling on-line processing of gaze stimuli and measurement of preferences for interpersonal distance directly from the distance that the participants maintained with the robot.

Another study by Mutlu et al. (2009) explored how a robot might use gaze cues to signal participation structure in a three-party conversation and how this structure shaped participants' conversational behavior. There were three conditions, which differed according to the percent of time the robot gazed at each of the two participants in that condition. In one condition, the robot looked exclusively at one participant (the *addressee*), signaling that the second participant had the role of *overhearer*. In the second condition, the robot looked mostly at the *addressee* and occasionally at the second participant, indicating a role of *bystander*. In the third condition, gaze was distributed equally between the two participants, indicating that both were *addressees*. The results from the study showed that the participants conformed to the norms of their signaled roles in their conversational behavior 97% of the time and rated their experience with the conversation and involvement in the group consistently with their conversational roles (e.g., feeling excluded in the *overhearer* condition). The simulated social interaction approach enabled precise control of the robot's gaze behaviors to signal specific participation structures and illustrated how individuals integrate a perception of the robot's behavioral cues and their world knowledge, particularly the norms associated with the participation structure of a conversation, to make ToM inferences about the conversational intentions of the robot and follow the norms associated with inferred intentions.

#### Perception of social cues

ToM inferences are also informed by perceptions of social cues such as gaze. The study by Mutlu et al. (2009) illustrates how ToM might be studied using simulated social interaction. Examples of simulation-based protocols also include studies that explore how the precise temporal and spatial congruency of such cues might affect outcomes such as joint attention, information recall, and task performance (Staudte and Crocker, 2011; Huang and Mutlu, 2012). Staudte and Crocker (2009) developed a video-based experimental paradigm in which a robot presented factual statements about objects in the environment such as “the sphere is next to a pyramid” and the robot looked toward the referenced objects (e.g., sphere and pyramid) 800–1000 milliseconds before the object names were spoken. The study manipulated the congruency between the robot's linguistic and gaze references and showed that participants confirmed the correctness of the statements faster when the two references were congruent. The simulation-based approach not only ensured that gaze cues were presented with precise timings but also enabled the presentation of incongruent cues in gaze and speech that is difficult to reliably produce by human confederates in an interactive protocol.

**Figure 6:**
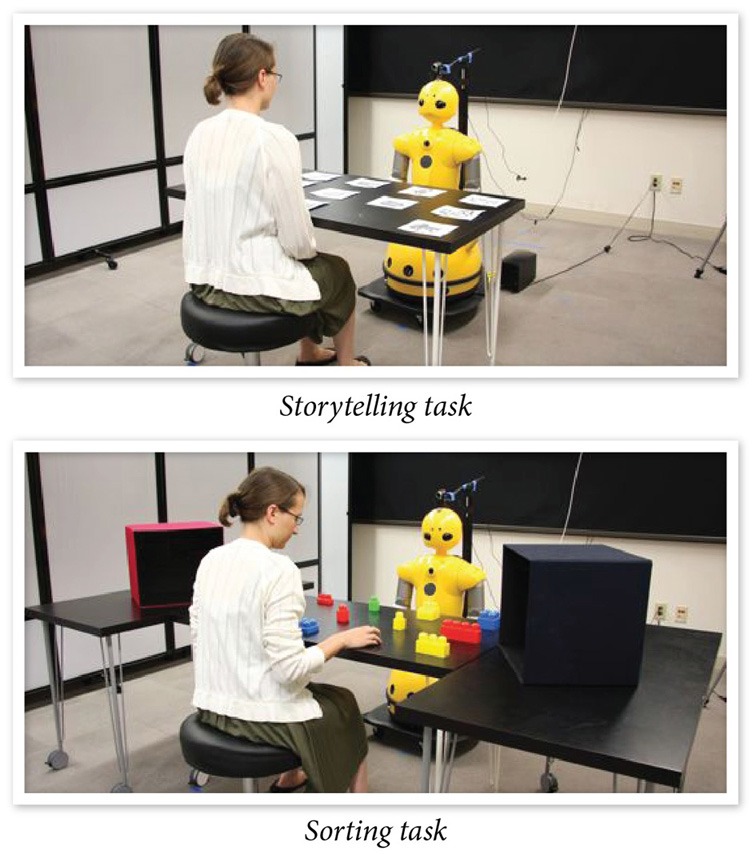
**The robot is using gaze cues to establish joint attention with the participants in a storytelling task (top) and a sorting task (bottom) (Huang and Mutlu, 2012)**.

Another study by Huang and Mutlu (2012) extended these results by comparing congruent gaze and speech cues to temporally incongruent cues by introducing a delay into the robot's gaze shifts such that they were produced 500—1000 milliseconds after the onset of linguistic references. The study also contextualized these comparisons in two realistic tasks. The first task involved the robot recounting a story and referring—using linguistic and gaze cues—to a set of props. In the second task, the robot provided instructions to participants to complete a sorting task, referring to objects to be sorted using linguistic and gaze cues (Figure 6). The results from the study showed that participants recalled the details of the story better in the first task than the second task, and were faster at locating to-be-sorted objects when the robot used congruent speech and gaze cues than when cues were spatially or temporally incongruent. In addition to extending the results obtained by Staudte and Crocker (2009) to a physically situated paradigm, this study demonstrated the role of temporal congruency in ToM processes. The study also illustrated the effects of perception of social cues on the outcomes of ToM processes such as information recall and task performance in joint action. When the robot used congruent gaze cues, participants established stronger associations between objects in the environment and verbal information presented by the robot, thereby showing improved task performance.

#### Interpretation of actions

**Figure 7:**
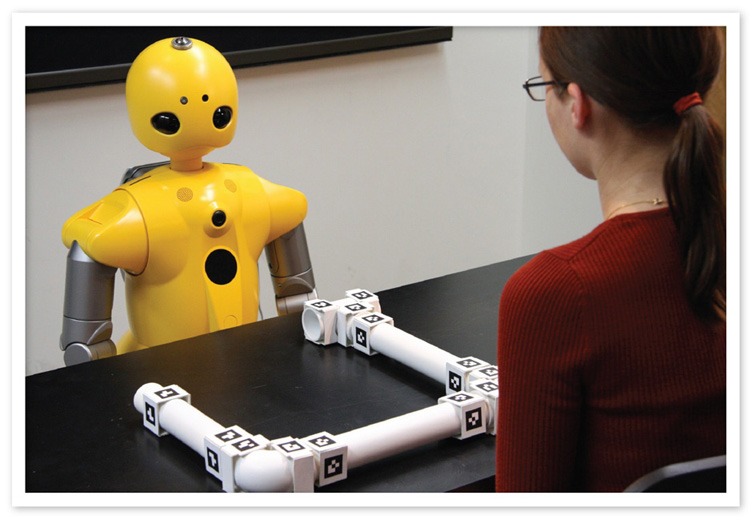
**The robot combines cognitive simulation and simulated social behaviors to maintain a model of the participant's progress in the task using action observation and to provide feedback using conversational repair (Mutlu et al., 2013)**.

Mechanisms such as action observation (Knoblich et al., 2011) also facilitate ToM processes by establishing a “procedural common ground” between interaction partners (Clark, 1996). Paradigms developed to study this mechanism most commonly follow the cognitive simulation approach (e.g., Trafton et al., 2005; Breazeal et al., 2006) or employ a coupling of the simulated social interaction and cognitive simulation (e.g., Gray et al., 2005; Bicho et al., 2011). A recent study used a paradigm in which a robot provided participants with instructions on how to assemble a structure and, by observing the participant's actions, maintained an internal model of the participants' progress, specifically modeling potential breakdowns in the participant's understanding or execution of the instructions (Mutlu et al., 2013) (Figure 7). When the robot inferred misunderstandings or confusion from its observations of participant actions or lack thereof, it offered clarifications on the actions that the participant must take to successfully progress in the task using conversational repair strategies (Clark, 1994). Compared to the robot only providing instructions, the robot that made ToM inferences through action observation and offered clarifications based on these inferences received fewer requests for help from the participants when breakdowns occurred. This protocol illustrates how the cognitive simulation and simulated social interaction approaches might be combined to create truly interactive experimental paradigms in which participants and artificial agents employ ToM mechanisms. Cognitive simulation enabled the robot to use its observations of participant actions to infer misunderstandings and confusions and to plan appropriate actions to offer clarifications. The simulated social interaction approach established communication between the participant and the robot and enabled the robot to use conversational strategies to execute its plan to offer clarifications.

### Limitations of computational paradigms

Simulation-based approaches offer unprecedented methodological advantages for studying social cognition and ToM processes in particular. They offer greater experimental control, on-line processing of stimuli, a richer set of measures for ToM outcomes, and truly interactive experimental scenarios, enabling the study of complex interactions and affording more ecologically valid research. These approaches, however, build on a number of assumptions and are affected by a number of factors that limit their promise.

#### Differences in simulated and real interactions

A key assumption that the simulation-based approaches discussed above make is that simulations match “real” stimuli and that interactions with artificial agents are equivalent to interactions with humans. While theoretical accounts such as the mindlessness hypothesis (Langer, 1992; Nass and Moon, 2000) and the findings presented above suggest that simulated interactions closely approximate human interactions, a critical examination of the differences between these forms of interactions is essential.

#### Design of computational platforms

Another factor that might limit the generalizability of findings obtained in simulated approaches is the design of the simulation platform. For instance, the experimental protocol developed by Mutlu et al. (2009) to study leakage gaze cues employed two robot platforms to investigate whether the design of the specific robot platform used in the study affected these inferences. The results showed that leakage gaze cues elicited ToM inferences, thus improving participant performance in guessing the robot's pick, when presented by a highly humanlike android but not when presented by a robot with a stylized design that featured cartoon-like features (although a trend in the data toward an effect was present). This finding suggests that the physical design of the robot platform might affect social cognition and ToM inferences with artificial agents and that different designs might vary in the extent to which they activate ToM mechanisms. While the choice of experimental materials and the design of experimental stimuli has been found to affect social cognition in conventional experimental paradigms, such as the attractiveness of faces shown in stimuli interacting with gaze manipulations (Kampe et al., 2001), a better understanding of how design features for artificial agents affect social cognition and the development of benchmark platforms for research in social cognition might help isolate such effects.

#### Simplifications in simulations

Simulations of cognitive and behavioral mechanisms necessarily involve simplifications in representation and presentation due to difficulties in capturing the complexity of these mechanisms. Such simplifications might result in artificial agents displaying “robot-like” behaviors that fail in activating ToM mechanisms or eliciting feelings of eeriness or discomfort—an effect often referred to as the “uncanny valley” (Mori, 1970). Recent findings indicate that exposure to artificial agents results in a shift in what participants consider to be acceptable or preferred human qualities (Chen et al., 2010), suggesting that properly acclimating participants to simulations might alleviate such effects or that people might attune to simplified behaviors as artificial agents become commonplace.

#### Individual differences

A final consideration in the use of simulation-based experimental paradigms is individual differences in responses to artificial agents. Studies to date have identified individual differences based on participant sex (Mutlu et al., 2006), cultural background (Nomura et al., 2008), personality (Walters et al., 2008), and pet ownership (Mutlu et al., 2009). While such differences might also be observed in conventional experimental paradigms, whether these differences predict those in simulation-based paradigms is unknown. Simulation-based studies of ToM processes must consider demographic and attitudinal factors to establish a better understanding of their effects.

## Summary and conclusions

The study of ToM has provided much knowledge regarding how the ability to reason about mental states develops in typical children and insight into impaired ToM in clinical populations. The tasks developed through this research largely measure individuals' abilities to utilize and integrate information from shared world knowledge, social cues, and physical actions to infer the mental states of others to predict future behavior. While the passive and reflective methods traditionally used to study ToM have been invaluable to understanding the mechanisms associated with the ability to infer mental states, we, with others (Risko et al., 2012), argue that investigations using interactive, on-line approaches to study ToM and social cognition hold great promise to enrich the established knowledge base. As described, emerging methods, including the use of virtual and robotic platforms, provide opportunities to study human behavior in social interactions while maintaining experimental control. These emerging interactive and computational methods may, in addition to extending knowledge regarding ToM processing, enable the development of innovative, technology-driven protocols for improving mentalizing in cases of disorder.

### Conflict of interest statement

The authors declare that the research was conducted in the absence of any commercial or financial relationships that could be construed as a potential conflict of interest.
